# ETU-Net: efficient Transformer and convolutional U-style connected attention segmentation network applied to endoscopic image of epistaxis

**DOI:** 10.3389/fmed.2023.1198054

**Published:** 2023-08-09

**Authors:** Junyang Chen, Qiurui Liu, Zedong Wei, Xi Luo, Mengzhen Lai, Hongkun Chen, Junlin Liu, Yanhong Xu, Jun Li

**Affiliations:** ^1^College of Information Engineering, Sichuan Agricultural University, Ya'an, China; ^2^Department of Otorhinolaryngology Head and Neck Surgery, Ya'an People's Hospital, Ya'an, China; ^3^Sichuan Key Laboratory of Agricultural Information Engineering, Ya'an, China

**Keywords:** epistaxis, nasal endoscope, deep learning, image segmentation, Transformer, attention mechanism, model fusion

## Abstract

Epistaxis is a typical presentation in the otolaryngology and emergency department. When compressive therapy fails, directive nasal cautery is necessary, which strongly recommended operating under the nasal endoscope if it is possible. Limited by the operator's clinical experience, complications such as recurrence, nasal ulcer, and septum perforation may occur due to insufficient or excessive cautery. At present, deep learning technology is widely used in the medical field because of its accurate and efficient recognition ability, but it is still blank in the research of epistaxis. In this work, we first gathered and retrieved the Nasal Bleeding dataset, which was annotated and confirmed by many clinical specialists, filling a void in this sector. Second, we created ETU-Net, a deep learning model that smartly integrated the excellent performance of attention convolution with Transformer, overcoming the traditional model's difficulties in capturing contextual feature information and insufficient sequence modeling skills in picture segmentation. On the Nasal Bleeding dataset, our proposed model outperforms all others models that we tested. The segmentation recognition index, Intersection over Union, and F1-Score were 94.57 and 97.15%. Ultimately, we summarized effective ways of combining artificial intelligence with medical treatment and tested it on multiple general datasets to prove its feasibility. The results show that our method has good domain adaptability and has a cutting-edge reference for future medical technology development.

## 1. Introduction

Epistaxis is a typical emergency for primary care physicians and commom presentation in otolaryngology and emergency department. According to statistics, more than 60% of the population has experienced epistaxis in their lifetime, and 6% need medical help (1). Epistaxis has a bimodal age distribution, common in children around 10 years old and older adults around 60 years old (2, 3). Epistaxis mostly shows as unilateral hemorrhage, and a few can occur as bilateral. Most anterior epistaxis originates primarily from the Kiesselbach plexus (3), whereas the posterior epistaxis is less common but more fierce. Local illnesses of the nasal cavity, such as dry mucosa, trauma, inflammation, tumors, etc., are the main causes of epistaxis. Systemic diseases, such as coagulation dysfunction, hypertension, acute febrile infectious diseases, etc., can also induce it (4).

Local compression and nasal spray with vasoconstrictor are the primary treatment options for epistaxis (5). When such methods are ineffective, directive nasal cautery by silver nitrate or electro-drying should be performed as soon as possible under the nasal endoscope (6, 7). Nasal endoscope is a long, rigid tube used to view the interior of the nasal cavity. Its camera at the front captures color high-definition video images projected to the screen in real-time (8). During the operation, to avoid local ulcers, mucosal atrophy, and nasal septal perforation caused by excessive damage to the nasal mucosa, the operator needs to accurately judge the shape of malformed vessels under the nasal endoscope. Due to the complex anatomical structure of the nasal cavity and the rich submucosal vascular network, it is a big challenge for primary care physicians and junior physicians to accurately identify malformed vessels. Now, the emergence of artificial intelligence image segmentation algorithms provides the possibility to solve this dilemma.

In recent years, with the development of deep learning technology, artificial intelligence combined with medical treatment has become a research hotspot (9). The deep learning algorithm has the ability to process a large amount of complex medical information and images by continuously accumulating and learning disease data. Taking image segmentation as an example, it refers to the classification of images at the pixel level. It separates the target and the background to perceive the target accurately. Yadav et al. (10) developed a probabilistic neural network based on deep learning to segment the brain tumor area from the image, achieving a segmentation accuracy of 99.21%. Dong et al. (11) achieved high performance in the automatic segmentation of coronary arteries with a multi-scale feature aggregation method. Bateson et al. (12) emphasized the passive domain adaptability of segmentation. They proposed a framework using a priori entropy minimization method so that the model can maintain better indicators in different segmentation tasks. While good results have been achieved in the field of image segmentation, there is still a lack of research on the problem of epistaxis treatment under nasal endoscope. This is further exacerbated by the difficulty in acquiring medical image data, which requires specialized equipment and involves ethical and privacy issues. These challenges present a significant barrier to research in the field of epistaxis treatment, including the development of artificial intelligence solutions.

Our work focuses on segmentation of epistaxis image under nasal endoscope and aims to use deep learning technology to help clinicians accurately judge malformed vessels. The contribution of this paper mainly has three aspects, which are summarized as follows:

(1) Collected and organized a Nasal Bleeding dataset, which can be used for segmentation model learning and evaluation in the field of epistaxis;

(2) Proposed the ETU-Net, a model which uses a U-style structure combining the convolutional neural network and Transformer, and can be used for segmentation tasks of medical images;

(3) Introduces the related strategies of model training, evaluates the performance of the proposed model on multiple datasets, and compares it with multiple advanced models.

## 2. Related work

### 2.1. Convolutional neural network for image segmentation

Convolutional Neural Network (CNN) (13) is a classic model that combines deep learning and image processing technology. As one of the most representative neural networks in deep learning technology, it has made many breakthroughs in image analysis and processing. Many accomplishments based on CNN have been realized, including image feature extraction and classification (14), pattern recognition (15), etc., in the widely used academic picture annotation set ImageNet (16). CNN is a deep model with supervised learning. Its basic idea is to share the weights of the feature map at different positions of the previous layer of the network and use the relative spatial relationship to reduce the number of parameters to improve the training performance. When operating image segmentation, CNN has excellent feature extraction capabilities and good feature expression capabilities. Unlike traditional image processing techniques, it requires manual image features extraction, and does not need too much preprocessing of images. Therefore, CNN has been widely used in medical image segmentation in recent years (17–19).

**Figure 1:**
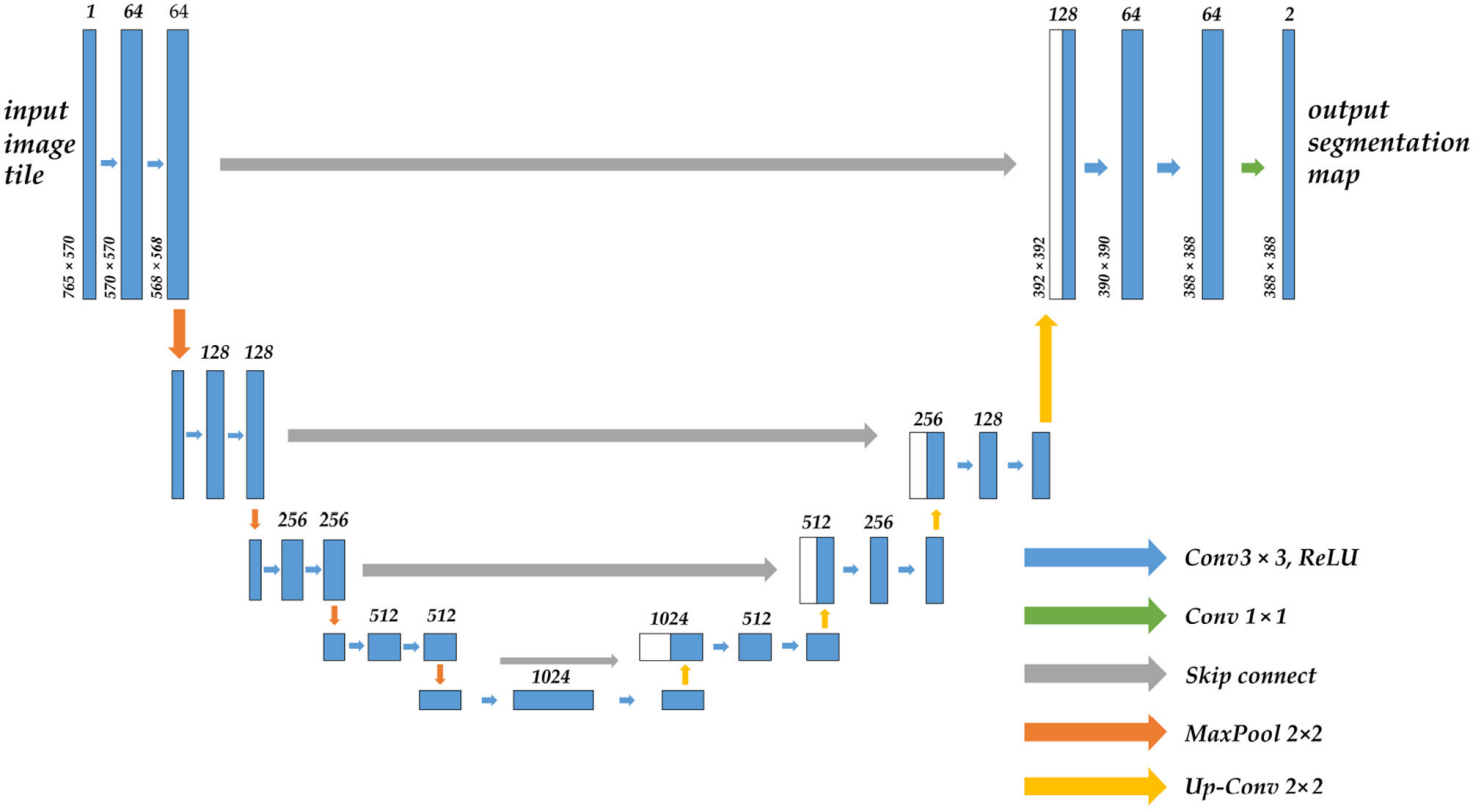
The structure diagram of the U-Net model, which uses the encoder-decoder architecture, the arrow in the middle is the skip connection, the upper part of the bar is the corresponding number of channels, and the left side is the size of the feature map.

To successfully apply CNN in image segmentation, the first issue must be addressed is that the fully connected layer at the conclusion of traditional CNN can only get one-dimensional category probability information, causing the lose of global pixel information. Therefore, in Long et al. (20) proposed FCN (full convolutional neural network), which replaced the original fully connected layer of one-dimensional vector in a form of a convolutional layer. It is a pioneering work in CNN image segmentation. U-Net (21) was born on the basis of FCN, which is dedicated to biomedical images. Its network adopts the U channel structure of the encoder-decoder, and the channels are connected by jumps, as shown in Figure 1. Similar improved network U-Net++ (22) and three-dimensional structure V-Net (23) are successively being proposed. CNNs that have demonstrated excellent capabilities in image segmentation also include DeepLab v3 Plus (24), PSPNet (25), and HrNet (26), but most of them lack global multi-scale recognition and context modeling capability and cannot pay attention to the feature and position information directly associated with the segmented object.

### 2.2. Transformer: a new deep learning network architecture

**Figure 2:**
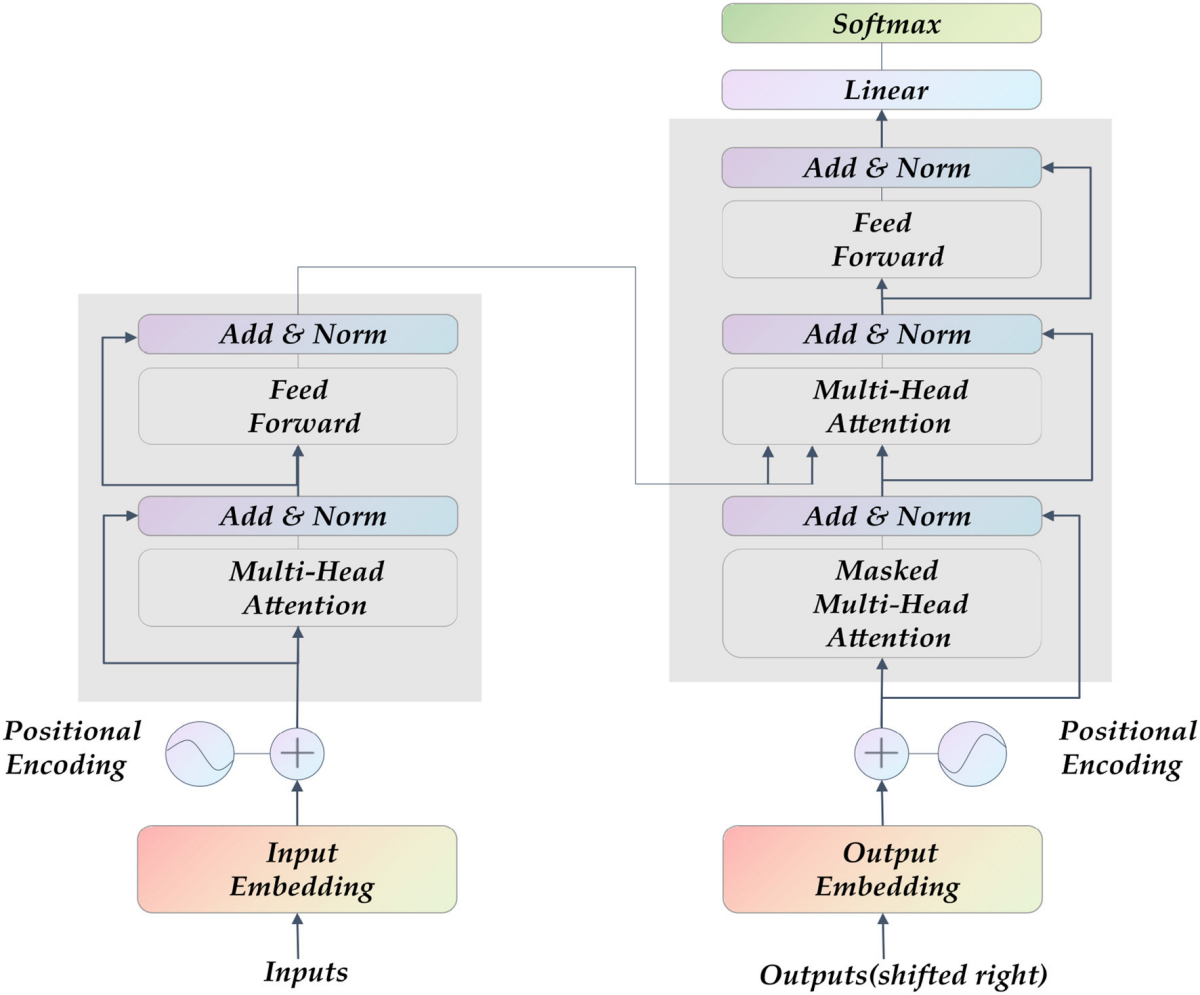
The Transformer model's structure diagram comprises a self-attention layer and a feed-forward neural network. Its input is sequence data, which is suitable for parallel computing.

Transformer (27) is currently the hottest network architecture in the field of artificial intelligence. It was proposed in 2017. The entire network structure of the Transformer is completely composed of the attention mechanism and the Multilayer Perceptron (MLP) feedforward neural network, as shown in Figure 2. Some CNNs also adopted attention mechanism, such as SE-Net (28) and CA-Net (29). They mainly draw on the attention allocation mechanism of human vision, and learns an information vector associated with the context through the module to assign weights to the input sequence. The MLP feedforward network is the so-called multi-layer perceptron, and the different layers are connected in a fully connected manner.

The classic Transformer uses a self-attention mechanism, which is an Encoder-Decoder superposition model. It was originally used for natural language processing in which sentences need to be segmented in the input network. Similarly, in image segmentation training, the image segmented into multiple patches for inputs, and mapped into a linear embedded sequence which is encoded by the encoder. Then the decoder decoded it by the output of the class embedding, recover the original image by up sampling and predict the corresponding class for each pixel to output the final prediction map. Segmenter (30) is a complete semantic segmentation model of the Transformer architecture. Experiments have proved that it captures the global context information of the image well and can be used for long-term modeling and encoding. Similar to Segmenter there is the Swin-Unet (31) model and the amazing point is that it completely replaces the convolution block in the original U-Net with Transformer, and it is 2.28% higher than U-Net in terms of dice score. However, the pure Transformer, despite its high precision and strong performance, does not perform well on small datasets, and at the same time, too many fully connected layers will bring a dramatic increase in the amount of calculations. Such simplistic model, which relies on storage space and processing resources for accuracy performance, is clearly inapplicable to medical image segmentation. TransUNet (32) has noticed this. It adopts a hybrid structure of CNN and Transformer, uses CNN to obtain feature maps and inputs them into Transformer for encoding, and uses a cascaded upsampler to ensure accurate prediction. Moreover, UCTransNet (33) starts from the channel attention mechanism, optimizes the skip connection in U-Net, and proposes a multi-scale channel crossing information module. Based on above, it is not difficult to find that the architecture of CNN combined with Transformer can be compatible with the advantages of both, and has achieved a new breakthrough in image segmentation, which has reference significance for the work of this paper.

## 3. Materials and methods

### 3.1. Dataset acquisition

**Figure 3:**
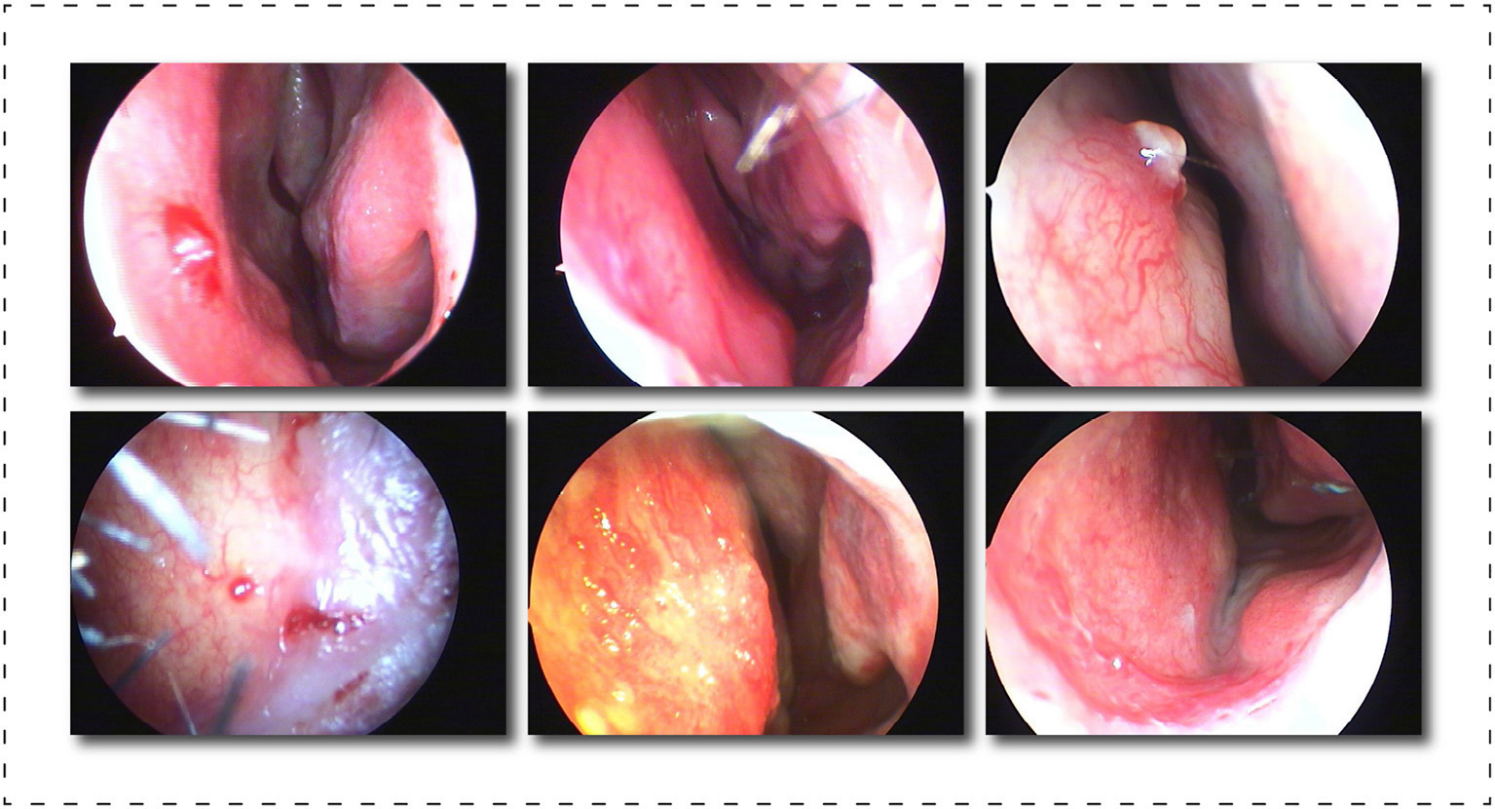
Display of part of the dataset, which includes, blurring, reflections, and spot and tendril vascular malformations.

Image segmentation is a pixel-level classification task, and training a model with superior performance and accurate recognition is closely related to the quality of the dataset. In existing reference sources, we could not find a professionally annotated epistaxis medical imaging dataset with corresponding segmentation masks. So we propose a dataset called Nasal Bleeding. This dataset utilizes epistaxis image data collected by professional nasal endoscopy equipment (OTV-SC, Olympus) since May 2020 at the Ya'an People's Hospital, Sichuan Province, China, and is used for the development of the deep learning segmentation model in this paper. These images were collected during nasal examinations of patients who voluntarily consented to the study. After the images were collected, all the data were desensitized to ensure that no ethics and personal privacy of patients were involved. There are a total of 405 images in the entire original dataset, and Figure 3 shows some of our dataset images. The curated dataset includes a variety of conditions during the examination, including blurred perspective, reflections, heavy nasal bleeding, and spot and tendril vascular malformations.

**Figure 4:**
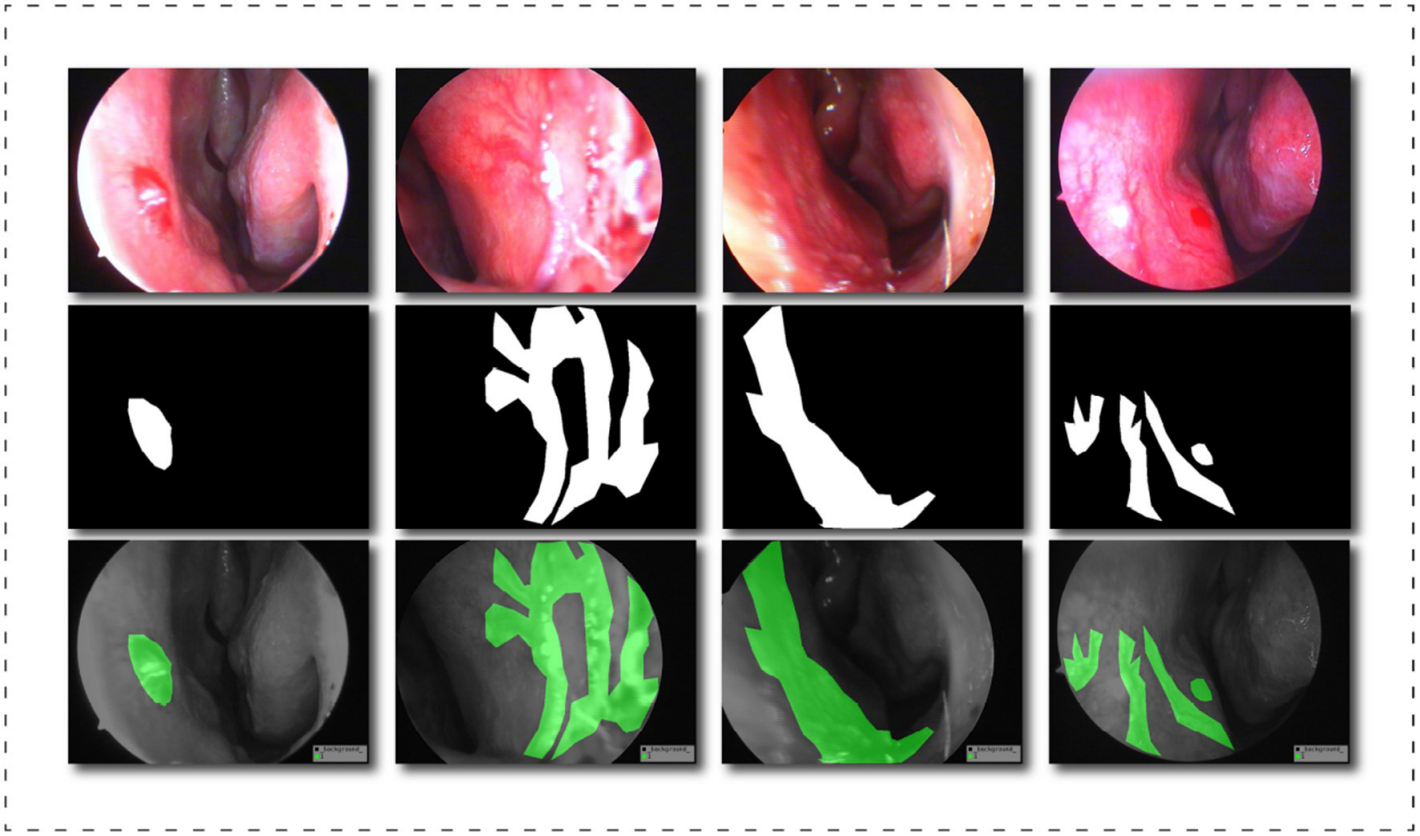
Schematic diagram of the dataset labels, from top to bottom are the original image, the black and white label image and the visualization result of the label in the original image.

After exporting the endoscopic epistaxis image, we saved the original RGB color image in JPG format (765 x 570, 24 bit, 960 dpi). The image labeling was completed by a professional rhinology physician with more than 5 years of specialist work experience. Using the Labelme image labeling tool based on the Anaconda prompt platform, all nasal endoscopic epistaxis images were labeled with bleeding sites and abnormal vessels, and were divided into two categories, spot or tendril vascular malformations, according to the shape of malformed vessels, and then cross-validated by two other rhinologists. If there is any dispute over the labeling of the epistaxis image, it will be decided by three people through joint discussion or voting. For annotating images pixel by pixel is both time and energy consuming, the strategy we adopt is similar to Curti et al. (34), and we try our best to focus on the bleeding areas. When malformed vessels are observed, we prefer to label those vessels rather than the whole bleeding area. After the annotation, a JSON file is obtained containing all the information about the image and the coordinate points used to generate the mask, for a total of more than thirteen million annotated pixels. Subsequently, we use the written Python script to generate masks in PNG format for each label. Finally, we got all the image datasets that meet the clinical segmentation requirements. This dataset is the first targeted dataset proposed on this issue so far, and it will help advance the research of deep learning in the field of epistaxis. In order to further explore the impact of label semantics on model performance, we divided the dataset into two cases. One uses two types of labels: background class and abnormal class; another uses three classes tags: background class, spotting tag, and bleed tag. Figure 4 shows some labels of our dataset.

### 3.2. Image preprocessing

We performed necessary image preprocessing procedures on the dataset, namely image improvement and data augmentation, to increase the practicability of the dataset as well as the robustness of the training model and its generalization ability. Common medical imaging datasets frequently contain uneven data distribution, a small number of image samples, and blurring. As a result, we first normalize the image using the following equation:


(1) $$\begin{array}{l} y=\frac{input-min\left(input\right)}{max\left(input\right)-min\left(input\right)} \end{array}$$

In Equation 1, input refers to the input pixel value of the image, output refers to the output pixel value of the image, max(input) and min(input) represent the maximum and minimum pixel values in the input, and finally this step can combine all pixel feature values are adjusted to the range of (0, 1), which will effectively prevent the influence of affine transformation and speed up the convergence of the model. In addition, we also manually refined the dataset to remove the influence of overly blurred images during model learning.

**Figure 5:**
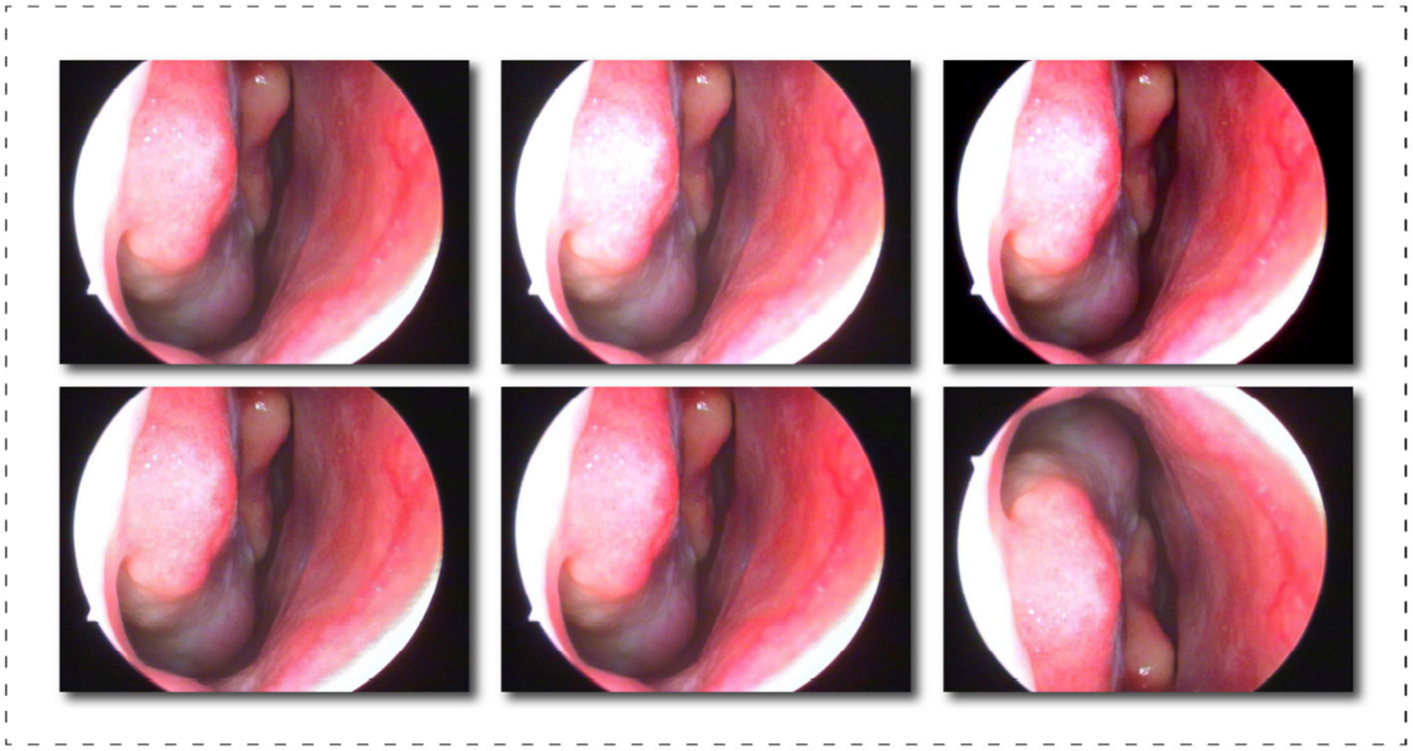
Schematic diagram of image preprocessing. From left to right and from top to bottom are the original image, adjusted brightness, adjusted contrast, adjusted sharpness, adjusted chroma, and random flip results. When the data is enhanced, the label will also change accordingly.

In this study, various data augmentation techniques were adopted, including random angle rotation, brightness modification, contrast enhancement, chroma sharpness enhancement, and mirror flip, to increase the dataset size to 3,582. Data augmentation has been employed to boost the dataset's diversity as well as geographic diversity expression. Modeling using augmented data is also expected to achieve significant robustness since certain augmented situations that may happen in the clinical practice can be captured. Additionally, effective solution(s) could be found for the overfitting issue arising from limited data. For example, as shown in Figure 5, a fragment of the image generated after image preprocessing, these techniques enhance dataset variability, which can help decrease the model's generalization error and improve its ability to recognize key features.

Random angle rotation can improve the model's ability to learn data from different angles, boosting the model's flexibility. Also, with the brightness modification and contrast enhancement, we adjust images' contrasts and brightness to improve their texture details, helping our model recognize essential features accordingly. Chroma sharpness enhancement could increase images' color saturation, improve their contrast, and highlight the target area in various lighting conditions, improving the data's quality. Flip is an efficient method to expand dataset size by mirroring images and improve recognition accuracy by enabling the model to identify the target from different perspectives.

To sum up, data augmentation is a feasible method to improve the quality and quantity of a dataset, including in medical imaging. By incorporating data augmentation techniques into the model, our model has become more robust and adaptable in clinical practice. This paper leverages these techniques to enhance the dataset's variability, which helps improve the model's performance and generalization.

### 3.3. Training parameters and evaluation metrics

The experiment described in this article was carried out on a machine running the Ubuntu 20.04 operating system. PyTorch 1.10.0 is the deep learning framework. The processor is an Intel(R) Xeon(R) Gold 5320 with a clock speed of 2.20 GHz and 32 GB of RAM. The graphics card is an NVIDIA RTX A4000 with 16 GB of video memory and Compute Unified Device Architecture (CUDA) 11.3 acceleration.

Our dataset is separated into training and testing sets in an 8:2 ratio. The training set is used for the model's continuous learning, and the test set is a batch of data that the model has never seen before to assess its generalization ability. The batch size of the model in each training is 12, the total number of training is 200 rounds, the initial learning rate is set to 0.0001, the learning rate is dynamically changed by the cosine annealing algorithm (35), the momentum is set to 0.975, and Adaptive Moment Estimation (Adam) (36) optimizer, which is a faster gradient descent optimizer, can comprehensively consider the first-order moment estimation and second-order moment estimation of the gradient, and calculate the update step size.

It is worth emphasizing that we selected a batch size of 12 due to our use of GPUs with a limited memory capacity of 24 GB. Our experimental experience suggests that a reasonably large batch size can lead to better results while still maximizing computing resources. We uniformly set the epoch to 200 rounds to ensure fairness. Furthermore, through prior research, we found that the loss function approached convergence around 200 rounds. Nevertheless, different models may have different convergence intervals, which prompted us to standardize the epoch of our model to 200 rounds after comprehensive consideration.

During the experiment, the model will continue to learn on the training set, and will eventually generate multiple weight results that perform well on the training set. In order to further evaluate on the test set, we design five indicators to comprehensively test the performance of the model from different aspects. The model with the highest comprehensive evaluation score among all the well-performed models is finally selected as the final application model. At last, we compared the designed ETU-Net model with various advanced models in current academia, and tested it on other medical datasets to reflect the advancement and domain adaptability of our model.

The intersection ratio refers to the ratio of the overlapping part of the two regions to the union of the two parts. It reflects the gap between the model segmented region and the real marked region. The more accurate the model prediction is, the Intersection over Union (IoU) value will be higher. The specific equation of IoU is as follows, where A represents the real label area and B represents the model prediction area. And mIoU is the average of different types of IoU.


(2) $$\begin{array}{l} IoU=\frac{A\cap B}{A\cup B},IoU\in \left[0,1\right] \end{array}$$

Precision refers to the proportion of true positive samples out of all the positive samples predicted by the model. Recall refers to the proportion of the positive samples predicted by the model out of all the true positive samples, that is, sensitivity. The specific equations of the two are as follows, where TP refers to the number of positive samples that are correctly classified. In this paper, this positive sample refers to the number of pixels that are correctly predicted as abnormal classes, and FP refers to the number of pixels that are predicted as abnormal classes but actually are the background class, FN refers to the number of pixels that are predicted to be the background class but actually are the abnormal class.


(3) $$\begin{array}{l} Precision=\frac{TP}{TP+FP} \end{array}$$

(4) $$\begin{array}{l} Recall=\frac{TP}{TP+FN} \end{array}$$

In evaluating a model's performance, the use of single metrics such as Precision and Recall may be inadequate, as high values in one metric may not give an accurate reflection of the overall performance of the model, particularly when compared to metrics with comparatively lower values. Consequently, we recommend the use of the F1-score equation to evaluate the model's performance across different tasks effectively, with a particular focus on image segmentation problems. The F1-score is obtained by computing the harmonic mean of Precision and Recall, yielding a comprehensive metric that can effectively evaluate a model's overall performance. High Precision scores indicate accurately detected object boundaries, while high Recall scores indicate that all object boundaries have been delineated accurately. Therefore, to achieve high performance in image segmentation problems, a model should strive for a balance between these two metrics. The F1-score equation provides a quantifiable measure and provides insight into how well the model is performing across these two metrics, making it an essential evaluation metric. The specific equation for the F1-score is as follows:


(5) $$\begin{array}{l} F1-score=\frac{2\times mPrecision\times mRecall}{mPrecision+mRecall} \end{array}$$

In this paper, we also introduce an average index of Dice-score, which is specially used to evaluate the pixel classification of abnormal classes, rather than the overall evaluation. The specific equation is as follows:


(6) $$\begin{array}{l} Dice-score=\frac{2\times TP}{2\times TP+FN+FP} \end{array}$$

### 3.4. Transfer learning and freeze-thaw training

There are obviously many similarities in the process of picture segmentation, such as the necessity to detect the contour of the object in the image, and the positions where the objects appear in the image are generally comparable. Based on this feature, we propose using transfer learning to boost the models learning ability. Transfer learning refers to the application of new learning tasks in an old field by reusing existing model learning weights, analogous to how humans learn new tasks. Transfer learnings major purpose is to train the model on a very big dataset to obtain appropriate weights, and then utilize the generated weight model as an initialized basic model to learn on a new dataset. We pass the experiment, proving that this method is feasible which can quickly promte the network's convergence and significantly improve the model's accuracy. It can be seen that objects in real life have many analogies, which is also in line with human perception. We chose the Pascal VOC 2007 dataset (37) as our prior knowledge for transfer learning, which is a large official dataset from the Pascal VOC competition, consisting of nearly 10,000 realistic images, including humans, animals, vehicles, and furniture categories.

Transfer learning can converge to a higher level of accuracy with less training data and less training time. We employ a freeze-thaw training strategy to use these priors more efficiently instead of randomly initializing the network. Since the features extracted by the backbone feature extraction part of the neural network on large datasets are common, in the early stage of network training, we can freeze the weights of the backbone network of the model without changing the parameters of the feature extraction network. The network is fine-tuned. In the middle and late stages of network training, in order to adapt to new learning problems, the entire network will be unfreezed to participate in model training, and relevant parameters will be greatly adjusted.

### 3.5. ETU-Net

**Figure 6:**
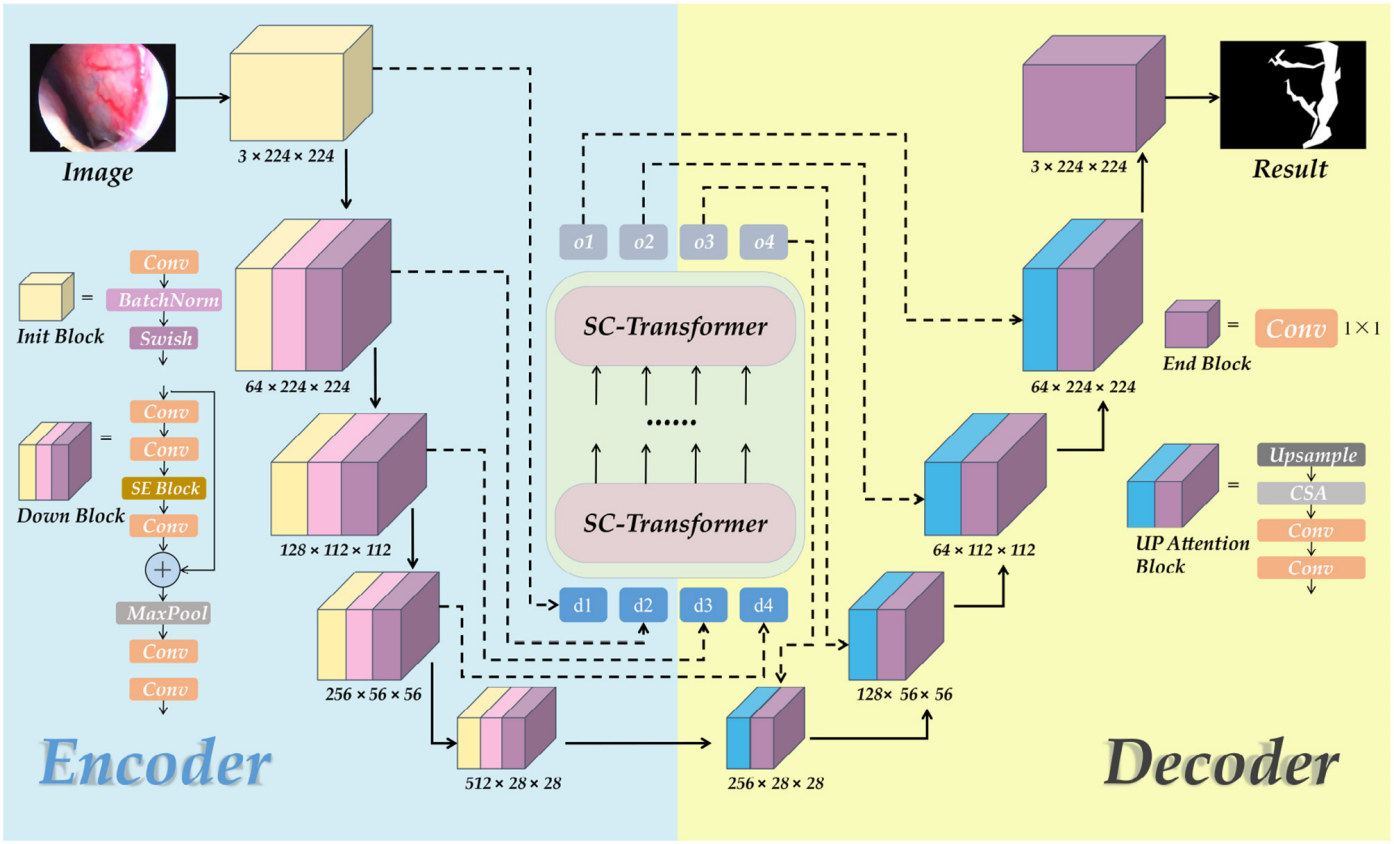
The structural diagram of the ETU-Net model. The model is divided into a decoder and an encoder. The SC-Trnasformer multi-scale feature fusion module connects the middle part. The input network is an original image of 3× 224×224. The corresponding prediction map will be output.

In this paper, we propose a network model named ETU-Net, whose structure is shown in Figure 6. The overall architecture of ETU-Net is in the form of Encoder-Decoder. The left side is the model encoder, which comprises an initialization block and four Down Blocks. It is mainly responsible for image feature extraction and will gradually reduce image feature information through the network layer. The right side is the model decoder, consisting of four Up Attention Blocks and one End Block, is mainly responsible for restoring the target details and spatial dimensions through the image features extracted by the encoder, and outputting pixel-level segmentation results one by one. Between the encoder and the decoder, we imitate the structure of U-Net and adopt a skip connection. This form can splice feature maps from the same encoder and decoder layer, and then restore the feature map produced during upsampling. Because the feature map now has more semantic information from the original image, it can increase image segmentation fineness.

Unlike the U-Net network, our model includes four contributions that offer equivalent methods for encoder, decoder, skip connection, and loss function to construct a novel U-paradigm network model, and an ablation experiment is devised to test its effectiveness. We will elaborate the ETU-Net network model in the following subsections.

### 3.6. Encoder

Encoders are composed of more efficient modules. The downsampling form of the original U-Net network is the module superposition form of the normal convolution-pooling-activation function. We design and propose the ETU-Net after referring to the structure of the constituent modules of EfficientNet v2 (38). The Down Block in EfficientNet v2 can well balance the three-dimensional features of depth, width, and resolution like the MBConv module in EfficientNet v2. At the same time, the Squeeze-and-Excitation (SE) attention module is embedded in the module, which naturally assigns attention weights to the feature maps during upsampling.

**Figure 7:**
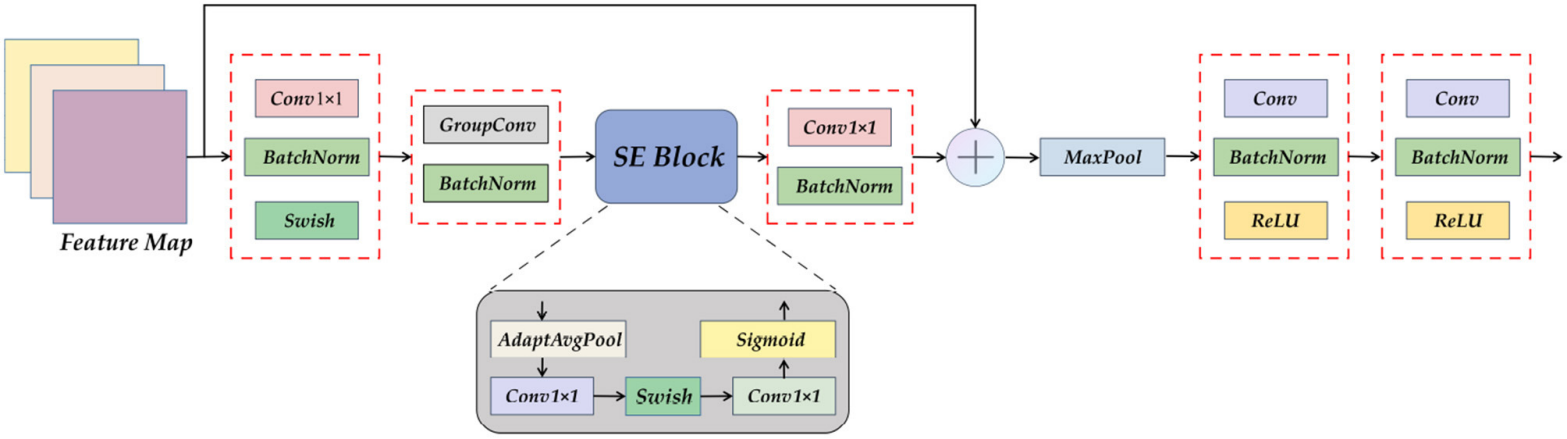
A detailed view of the Down Block module. It can be seen that Down Block uses convolution as a component, using attention mechanism, pooling, and residual connection.

**Figure 8:**
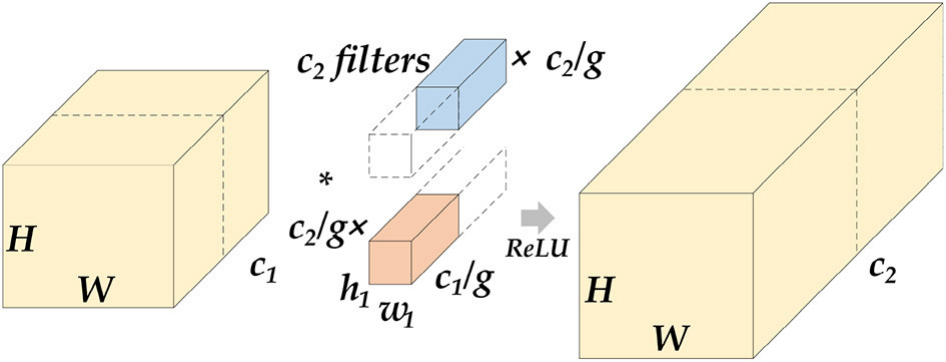
Schematic diagram of grouped convolution. We assume that the length and width of the feature map are H and W. After the group convolution operation, *c*_1_ represents the number of channels at the input, *c*_2_ represents the number of channels at the output, and g represents the number of groups. The input feature maps will be averaged into g groups by channel, perform conventional convolution on each group, and then concatenate the output feature map. The parameter amount of this operation will be reduced to $\frac{1}{g}$ of the original conventional convolution.

The Encoder part of ETU-Net consists of an initialization module and four Down Blocks. The initialization module is similar to the downsampling module of U-Net, but the activation function uses Swish (39). The schematic diagram of the composition of the Down Block is shown in Figure 7. It consists of two 11 convolution blocks, one group convolution, one SE Block, one max pooling, and two convolution blocks. The two 11 convolution blocks at the beginning and the end are used respectively for dimension increase and dimension reduction. The group convolution operation in the middle is different from ordinary convolution. Figure 8 depicts a schematic diagram in which the convolution kernels are dedicated to one channel and a channel is convolved by only one convolution kernel, which has the advantage of reducing the number of parameters during the deep convolution operation and improving the diagonal correlation between the convolution kernels. SE Block is a conventional attention module. Through the Squeeze process, that is, through AdaptAvgPool, the global compressed feature quantity of the current feature map is obtained, and then through the Excitation process, the weight of each channel of the feature map is obtained by two layers of full connection, and the dimension is reduced. Here we replace the fully connected layer with a 11 convolutional block equivalent, which can effectively reduce the amount of parameters. Because the traditional CNN network connection method frequently contains connections across neighboring layers, the more information is lost, the deeper the network. To avoid this problem, we use a shortcut connection method to connect multiple convolutional layers in series to form a deep network. That is, the shallow information can be mapped into the deep network to avoid gradient dispersion. At last, there are MaxPool layers and two convolutional layers, which expand the receptive domain of downsampling and preserve the texture features.

### 3.7. Skip connection

Transformer-based multi-scale feature fusion skip connection demonstrates that our proposed ETU-Net is a novel model that combines convolution and Transformer. CNNs and Transformers are two architectures commonly employed in the computer vision field, each possessing different strengths. CNNs excel at local feature extraction, while Transformers are suitable for global interaction and parallel computation. Based on such prior knowledge, we consider integrating the Transformer into the original U-shaped architecture for semantic segmentation networks.

In the original U-Net network, although skip connections are used between the same layers to alleviate the information loss that is prone to occur during the sampling process, it does not take into account the semantic gap between different layers, especially shallow encoders and the decoder. To achieve accurate medical image segmentation, the image features acquired by the decoder should contain information between layers. However, if more skip connections are added or a fully connected method is used, the network will become too redundant. Therefore, it can be considered to use the form of intermediate modules to perform feature fusion on the information obtained by skip connections, particularly leveraging the long-term dependency modeling ability of the Transformer.

**Figure 9:**
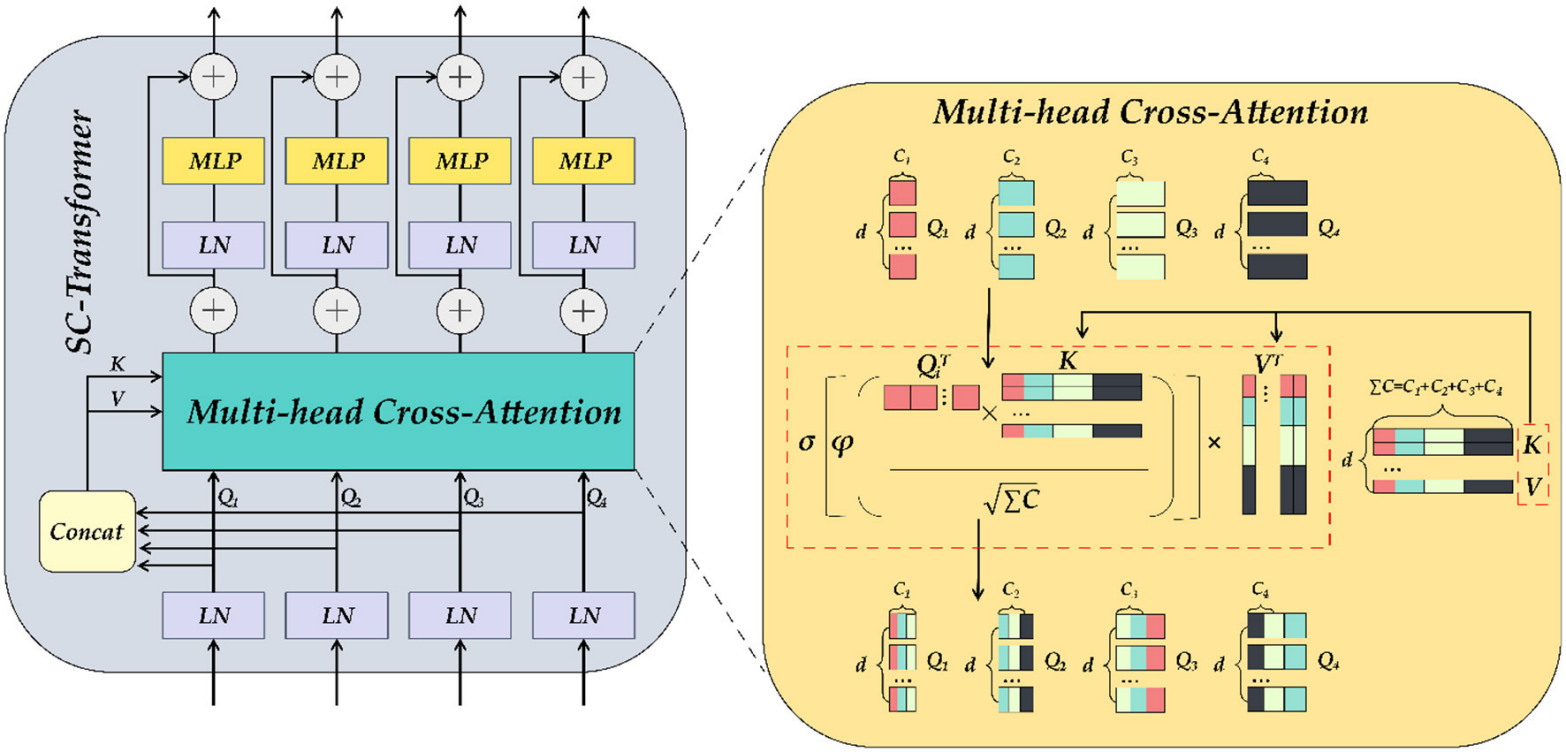
The detailed illustration of SC-Transformer is similar to Transformer. MLP and self-attention modules make up the SC-Transformer. The self-attention here is the multi-head cross-attention module.

We established the SC-Transformer in ETU-Net based on CCT (33), which consists of 4 Transformers, and each Transformer contains a multi-channel cross-attention, multi-scale feature embedding, and multiple MLPs, as shown in Figure 9.

First, the output of each downsampling layer is loaded into the SC-Transformer as input. Second, these input features will be dimensionally reduced into Transformers planarized sequence. Because there are a total of four layers of downsampling input, a total of four scales of encoder feature information can be obtained, and the number of channels remains unchanged during this process. Subsequently, the four Tokens (connected) are concat together, and each token is abbreviated as *T*_*i*_, and the Σ*T* after concat is then input into the multi-head cross-attention module as key and value, and then the encoding of each channel of the pairing as well as the interdependence between channels are completed by an MLP with residual structure, which refines the encoders correlation features. In the multi-head cross-attention module, there are five inputs in total, including four *T*_*i*_ as queries, Σ*T*_*i*_ after Concat as keys and values, and five outputs are weighted:


(7) $$\begin{array}{l} {\text{Q}}_{i}={\text{T}}_{i}W_{{\text{Q}}_{i}},\text{K}={\text{T}}_{\text{Σ}i}W_{\text{K}},\text{V}={\text{T}}_{\text{Σ}i}W_{\text{V}}\;\left(i=1,2,3,4\right) \end{array}$$

where $W_{Q_{i}}\in R^{C_{i}\times d}$, $W_{K}\in R^{C_{\text{Σ}i}\times d}$, $W_{V}\in R^{C_{\text{Σ}i}\times d}$, d refers to the length of the sequence, and *C*_*i*_ refers to the channel dimension of the four downsampling inputs. Using Q, K, and V, the similarity matrix can be generated through cross-attention, and the value can be weighted. The equation is as follows:


(8) $$\begin{array}{l} {\text{CA}}_{i}=M_{i}V^{⊤}=\sigma \left[\phi \left(\frac{Q_{i}^{⊤}K}{\sqrt{C_{\Sigma i}}}\right)\right]V^{⊤}\\ \;=\sigma \left[\phi \left(\frac{W_{Q_{i}}^{⊤}T_{i}^{⊤}T_{\Sigma i}W_{K}}{\sqrt{C_{\Sigma i}}}\right)\right]W_{V}^{⊤}T_{\Sigma i}^{⊤} \end{array}$$

Where σ is the Sigmoid activation function and ϕ the instance normalization approach (40), which normalizes the similarity matrix of each instance in order to smooth the gradient propagation. After the multi-head cross-attention module, the obtained MCA output is as follows, and the number of multi-heads in practical applications is *N* = 4:


(9) $$\begin{array}{l} MCA_{i}=\left(\text{C}{\text{A}}_{i}^{1}+\text{C}{\text{A}}_{i}^{2}+,\ldots ,+\text{C}{\text{A}}_{i}^{N}\right)/N \end{array}$$

(10) $$\begin{array}{l} {\text{O}}_{i}=\text{MC}{\text{A}}_{i}+\mathrm{MLP}\left(LN\left({\text{Q}}_{i}\right)+\text{MC}{\text{A}}_{i}\right) \end{array}$$

MLP refers to a multi-layer perceptron with a residual structure, and LN refers to a layer normalization method (41), which can normalize all features of each sample. The above equation will be repeated four times to build a four-layer Transformer, and the final output result will be concatenated with the previous layer's decoder output in the Up Attention Block module.

### 3.8. Decoder

**Figure 10:**
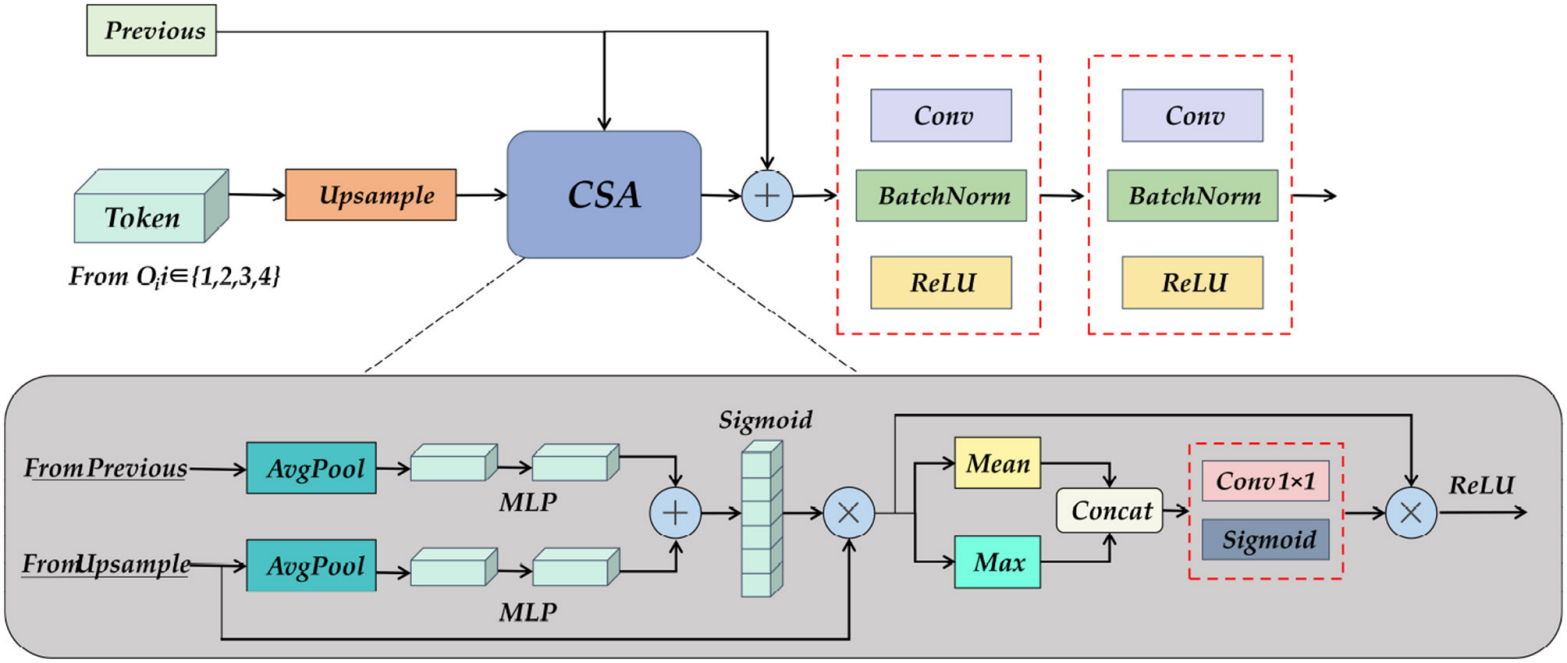
A detailed diagram of the Up Attention Block module. It consists of pooling, MLP, and spatial and channel attention modules, which reshape the information of the Transformer, and at the same time obtain the feature map of the previous layer and the output fusion of the skip connection.

To further obtain the semantic information obtained from the encoder and SC-Transformer, we designed the UP Attention module, as shown in Figure 10. Its primary task is to re-upgrade the Token formed after the feature map is loaded into the Transformer to a multi-channel feature map, followed by assigning corresponding weights to each feature using spatial and channel attention, and finally upsampling to restore image details and output the pixel class of the object.

The input of the UP Attention module is the feature map from the previous layer and the Token obtained by the SC-Transformer. The two are inconsistent in dimension, so the CSA module is needed to eliminate the ambiguity. The specific operation is to combine the two after average pooling and multi-layer perceptron, and then pass the activation function to obtain a multi-scale fusion feature map. Do matrix multiplication between the feature map and the upsampling result to obtain the attention distribution in the channel direction, and then calculate the mean value and maximum value of the results respectively, concatenate the two results to increase the dimension, and then multiply the result with the matrix, and finally get the weight feature map after multi-scale channel and spatial attention distribution through the activation function. The resulting output by the CSA module will also be spliced with the feature map of the previous layer, and the shape and size of the feature map will be gradually restored after two layers of convolution operations. Finally, a 3×224×224 prediction map is obtained through the End Block.

### 3.9. Loss function

By observing the pixels of the dataset, it is not difficult to find that our Nasal Bleeding dataset has a strong "long tail effect," that is, the distribution of the dataset is uneven, and the background class labels in the image occupy most of the label information. The network fends to pay more attention to improving the accuracy of the background class and ignore the abnormal class labels we really need, so we introduce the Focal Loss function (42), which assumes that the outliers and learning saturation are ignored. In some cases, the data that are easy to classify during the training process actually have little effect on improving the model, so the model should focus on those few hard-to-classify samples. This assumption fits well with our dataset distribution.

The function equation of Focal Loss is as follows. Among them, α and γ are adjustable factors that are artificially set. In the experiment, we set them as 0.5 and 2, and y = 0,1 represents two categories, which can be regarded as background and abnormal categories in the task of this paper. In the equation, *p* is the similarity between the model prediction result and the real label, and the larger *p* is, the more consistent the model prediction result is with the real label, that is, the closer the pixel classification is to category y. For samples with correct classification, the closer *p* is to 1, (1−*p*)^*r*^ tends to 0; for samples with inaccurate classification, *p* tends to 0, and (1−*p*)^*r*^ tends to at 1. The smaller *p* is, the more difficult it is to classify the sample. At this time, the adjustable factor can be used to assign greater weight to this sample, so that the entire function is optimized to favor these difficult samples, thereby improving the classification accuracy of the sample.


(11) $$Focal\;Loss=\left\{ \begin{array}{l} \;-\alpha {\left(1-p\right)}^{\gamma }\log\left(p\right),\text{if }\;y=1\\ -\left(1-\alpha \right)p^{\gamma }\log\;\left(1-p\right),\text{ if }\;y=0 \end{array}\right.$$

## 4. Results and discussion

### 4.1. Comparison with state-of-the-art methods

To demonstrate the segmentation performance of the ETU-Net proposed in this paper, we performed a comparative validation on the nosebleed dataset. The advanced models for comparison include: U-Net (21), DeepLabv3 Plus (24), SegFormer (43), PSPNet (25), and HrNet (26). The results of the experiment are visualized in Figure 10 and Table 2. The models mentioned in this section all adopt the method of transfer learning on the VOC2007 dataset, so the results obtained are the best metrics achieved by these models on the Nasal Bleeding dataset.

**Figure 11:**
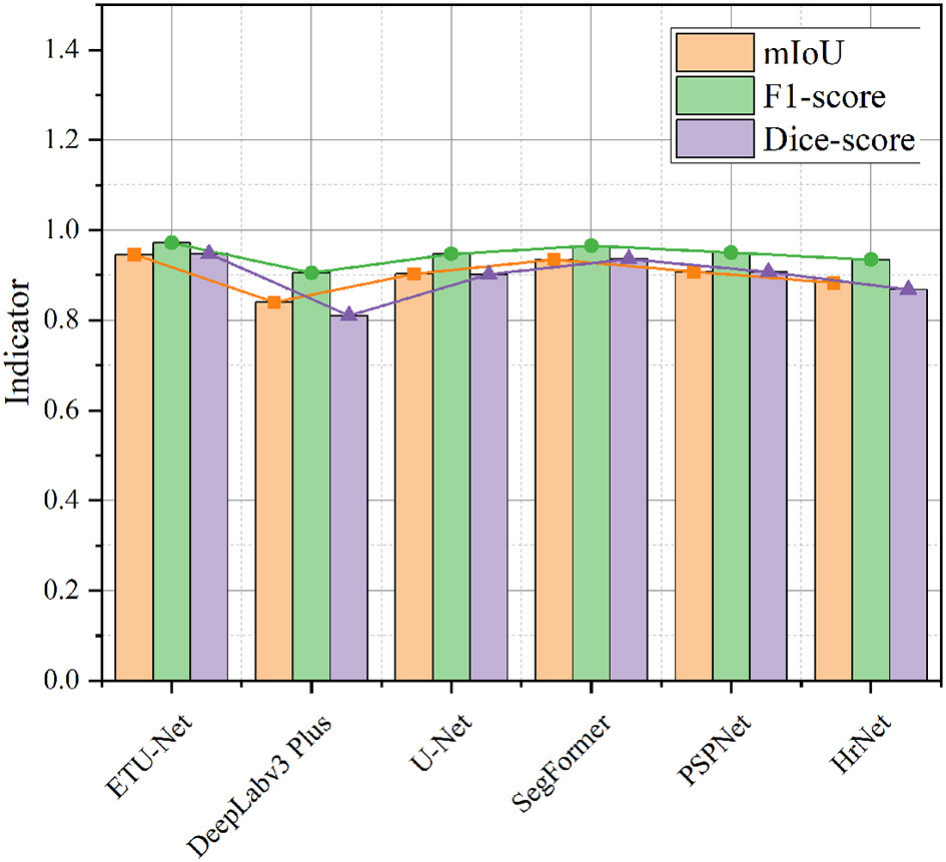
A line-column plot of the model results. The figure visually reflects the test performance of the six models on the Nasal Bleeding dataset.

First, as shown in Figure 11, we compared the performance of the six models on three indicators. It can be seen from the visualization that the mIoU and Dice-score of most of the models exceed 0.9, and the F1-score reaches above 0.9, indicating that the Nasal Bleeding dataset has good versatility, and there is no substantial decline in the indicators of a certain model on this dataset. Therefore, the Nasal Bleeding dataset can contribute to future model research on nasal bleeding image segmentation.

In Table 1, we further show the specific values, and supplement the average Precision and average Recall data of the six models. In the table, we also bold the indicators of the highest performance of the model. ETU-Net performed the best among these six models. Its mIoU and Dice-score reached 94.57% and 0.9473, which has a greater performance breakthrough than other advanced models. ETU-Net reached 97.38 and 96.93% on mPrecision and mRecall. In order to balance the two, the concept of the F1-score was introduced. It can be seen that the F1-score of ETU-Net is still 2.62% higher than the original U-Net, and the Dice-score has reached more than 5% improvement. DeepLabv3 Plus does not perform well on our dataset because the backbone of its network is a lightweight MobileNet. Although its inverted residual module can use depthwise separable convolutions to reduce computational overhead in high dimensions, it may cause the gradient return jitter during the optimization process, which will eventually affect the model performance. The second comprehensive ranking in each indicator is SegFormer, which is also a work of CNN combined with Transformer. Its encoder is a complete Transformer, while decoder is constructed by CNN. Similar to ETU-Net, SegFormer considers multi-scale feature fusion. Experiments have proved that this method is effective. Its mIoU reached 93.44%, F1-score and Dice-score reached 0.9652 and 0.9359. The PSPNet network uses the spatial pyramid pooling module to obtain contextual information and multi-scale information. The most obvious feature is that the input feature maps from different regions are pooled together and then bilinearly upsampled and spliced to the next layer. In the experimental results of the PSPNet network, the mIoU, F1-score, and Dice-score were 0.9073, 0.9497, and 0.9071, respectively. Based on the performance of the above models, we can conclude that the models using CNN combined with Transformer perform better in this dataset, because they can take into account the advantages of both, and perceive local and global information better. Similarly, the multi-scale fusion module helps to improve the performance of the CNN architecture, usually using a pyramid structure or a skip connection. To further lessen the amount of processing in the model, utilizing grouped convolution or separable depth convolution is a viable strategy.

**Table 1:** A data table of model results.

**Model**	**mIoU**	**mPrecision**	**mRecall**	**F1-score**	**Dice-score**
U-Net	0.9023	0.9455	0.9480	0.9467	0.9016
DeepLabv3 Plus	0.8396	0.9219	0.8889	0.9050	0.8102
SegFormer	0.9344	0.9644	0.9661	0.9652	0.9359
PSPNet	0.9073	0.9480	0.9515	0.9497	0.9071
HrNet	0.8827	0.9588	0.9110	0.9343	0.8678
ETU-Net	**0.9457**	**0.9738**	**0.9693**	**0.9715**	**0.9473**

The highest data in the figure are presented in bold.

### 4.2. Exploration of dataset semantic labels

In this section, we focus on exploring the impact of dataset semantic labels on the model training process to illustrate the evaluation results of using dataset 2 as the model in this paper. We use three models of U-Net, DeepLabv3 Plus and HrNet for experiments. It can be seen intuitively from the Table 2 that the results of the two types of datasets are generally higher than the results of the three types. Taking U-Net as an example, in the two cases, the difference between mIoU is 6.18%, and the difference between F1-score and Dice-score is 0.0387 and 0.0726. DeepLabv3 Plus is least affected by the label semantics of the dataset, and the change of most indicators is about 3%, and even the Dice-score on the three types of datasets is 0.02 higher than that of the two types of datasets. In general, the semantic labels of the dataset have an impact on all indicators of the model, especially mIoU. This is because mIoU is the similarity of the comparison area. Once the label information is refined, it will cause confusion among various classes, and may also be limited by the long-tail effect in the dataset, which leads to the model having a high degree of confidence in the categories with dominant numbers in the dataset, but low in those with inferior numbers, resulting in unfair segmentation results. This paper believes that we can try to improve these situations by expanding the dataset, increasing the weight of the loss function on the category, or resampling the inferior category. Due to the low demand for judging the type of epistaxis in the clinical practice operators only need to know which areas in the nasal cavity have bleeding symptoms, or find out abnormal vessels. Therefore, the dataset can be divided into background and abnormal categories to meet the needs of clinical diagnosis assistance. On the other hand, the datasets of the two types of labels in the experiment have a good influence on the model, so our subsequent experiments mainly use the two types of labels as reference results.

**Table 2:** The result table of the model under different categories of datasets.

**Model**	**Classes**	**mIoU**	**mPrecision**	**mRecall**	**F1-score**	**Dice-score**
U-Net	Two	0.9023	0.9455	0.9480	0.9467	0.9016
	Three	0.8405	0.9210	0.8953	0.9080	0.8290
DeepLabv3 Plus	Two	0.8396	0.9219	0.8889	0.9050	0.8102
	Three	0.7906	0.8871	0.8681	0.8774	0.8302
HrNet	Two	0.8827	0.9588	0.9110	0.9343	0.8678
	Three	0.8134	0.8975	0.8878	0.8926	0.8135
ETU-Net	Two	0.9457	0.9738	0.9693	0.9715	0.9473
	Three	0.8699	0.9342	0.9228	0.9285	0.8793

The classes column in the table represents the category division of the dataset, two indicates that there are only abnormal and background categories, and three indicates that there are background, tendril, and point categories.

### 4.3. Impact of transfer learning on model performance

As mentioned earlier, we adopted a model training method of transfer learning, which refers to using the prior knowledge of the model obtained on a large-scale dataset, and then training the model on different task datasets of the downstream branch. This method can often achieve good results, because the model has acquired the ability to obtain information like the outline texture of the perceptual image target, rather than randomly guess the results at the beginning of random initialization. We mainly use five models for comparison of transfer learning, as shown in the Table 3. Before transfer learning, the mIoU of each model under the same conditions was mostly around 83%, but after transfer learning, mIoU mostly reached more than 90%, an increase of nearly 7%. On the ETU-Net, after transfer learning, F1-score and Dice-score have increased respectively by 5.76 and 11.49%. Comparing this result with U-Net and SegFormer, we can found that transfer learning does not improve them as much as the ETU-Net. The mIoU of U-Net differs by 6.18% before and after, and the F1-score and Dice-score are improved 4.26 and 8.76%. SegFormers performance before transfer learning is not outstanding in all models. It is precisely because of transfer learning that mIoU has increased by 10.63%. It can be seen that transfer learning can improve the performance of the model in general, especially for CNN combined with Transformer architecture. Because Transformer lacks inductive bias for image space translation invariance and local relations, it frequently requires huge amounts of data to train, hence transfer learning on large-scale datasets is a crucial training approach for models of this architecture.

**Table 3:** Results table of transfer learning.

**Model**	**Transfer learning**	**mIoU**	**mPrecision**	**mRecall**	**F1-score**	**Dice-score**
U-Net	No	0.8405	0.9210	0.8953	0.9080	0.8290
		Yes	0.9023	0.9455	0.9480	0.9467	0.9016
DeepLabv3 Plus	No	0.7776	0.8499	0.8653	0.8575	0.7163
		Yes	0.8396	0.9219	0.8889	0.9050	0.8102
HrNet	No	0.8366	0.9467	0.8663	0.9047	0.8058
		Yes	0.8827	0.9588	0.9110	0.9343	0.8678
SegFormer	No	0.8281	0.8917	0.9075	0.8995	0.8146
		Yes	0.9344	0.9644	0.9661	0.9652	0.9359
ETU-Net	No	0.8570	0.9167	0.9205	0.9186	0.8497
		Yes	0.9457	0.9738	0.9693	0.9715	0.9473

This table details the performance changes of the five models before and after transfer learning.

### 4.4. Ablation experiment

In order to explore the influence between the various components of our proposed ETU-Net model, we conducted an ablation experiment and tried to split each component to study the relationship between them. Since SC-Transformer and the subsequent UP Attention Block have a fixed conversion of data types, the two cannot be independently formed into a block, so we regard the two as a component for research, and get four groups of comparison results, as shown in the Table 4. In the first row of data, the U-type network without Down Block and SC-Transformer has a large decline in model performance, and mIoU, F1-score and Dice-score, compared with the optimal combination, are reduced by 4.34, 2.62, and 5.07%, respectively. By introducing the Down Block model, the model get the ability to match weights with attention, and can filter useless information during the downsampling process, therefore, compared with the original situation, mIoU has increased by 2.79%, and Dice-score has increased by 3.35%. If only SC-Transformer is added to the model, although the Dice-score index has increased by 4.53%, the mIoU has decreased because of the lack of a powerful downsampling module. Even if the multi-scale fusion of each layer is performed, the image features obtained initially are not as good as those obtained by the Down Block module. It can be concluded from the data table that each module has various contributions to the final performance of the ETU-Net model, and only when the modules are used in combination can the optimal experimental results be achieved.

**Table 4:** Ablation experiment result table.

**Model**	**DB**	**SCT**	**mIoU**	**mPrecision**	**mRecall**	**F1-score**	**Dice-score**
ETU-Net	No	No	0.9023	0.9455	0.948	0.9467	0.9016
		Yes	No	0.9302	0.9566	0.9604	0.9585	0.9318
		No	Yes	0.8930	0.9476	0.9393	0.9434	0.9424
		Yes	Yes	0.9457	0.9738	0.9693	0.9715	0.9473

In this table, DB indicates whether there is a Down Block module, and SCT indicates whether there is an SC-Transformer and Up Attention Block module.

### 4.5. Exploring the domain transferability of models

As shown in the Table 5, we explored the domain transferability of the ETU-Net model in three other different medical research fields, namely DRIVE (44), Kvasir (45), and Liver (46) dataset. In general, ETU-Net is suitable for these fields, and compared with the optimal results, the performance of ETU-Net is not behind or even surpassed. DRIVE is a classic retinal blood vessel image segmentation dataset, consisting of 40 JPEG color fundus images, including seven abnormal pathological cases. On this dataset, we compared with the other three models, and ETU-Net is close to the most advanced RV-GAN (47) on most indicators, and surpasses it in sensitivity by 4.29%, with only 0.0036 difference in F1-score. Our model is also tested on the Kvasir dataset, an open dataset of gastrointestinal polyp images, which is often cited by deep learning models for disease segmentation in recent years. The results show that our model performs best on the three indicators proposed by the dataset. Compared with the previous best model FCBFormer, the Dice-score exceeds its 1.58%. Compared with U-Net, Dice-score increased by 21.89%. The overall mIoU has reached the best level, which is 95.02%. On the Liver dataset, a liver tumor segmentation benchmark containing a total of 200 CT scan images, ETU-Net also performed the best. Compared with the model indicators already given, the mIoU of ETU-Net increased the most, reaching 9.42%, and mPrecision and mRecal respectively reached 99.34 and 99.39%. Therefore, the ETU-Net proposed in this paper can be further extended to other fields of medical segmentation. Whether it is endoscopic shooting or CT scan data, the model has good adaptability and is less dependent on the type of data source.

**Table 5:** Performance evaluation tables on three datasets.

		**Model**	**F1-score**	**Sensitivity**	**Specificity**	**Accuracy**
		U-Net	0.8174	0.7822	0.9808	0.9555
		DFUNet	0.8190	0.7863	0.9805	0.9558
		RV-GAN	**0.8690**	0.7927	**0.9969**	**0.9790**
	DRIVE	ETU-Net(Ours)	0.8654	**0.8356**	0.9707	0.9620
		**Model**	**Dice-score**	**mIoU**	**mRecall**	
		U-Net	0.7821	0.8141	0.8450	
		ResUNet++	0.8074	0.7231	0.7874	
		FCBFormer	0.9385	0.8903	0.9401	
	Kvasir	ETU-Net(Ours)	**0.9533**	**0.9502**	**0.9733**	
		**Model**	**Dice-score**	**mIoU**	**mPrecision**	**mRecall**
		U-Net	0.9302	0.8985	0.9279	0.9429
		Res-U-Net++	0.9219	0.8898	0.9119	0.9428
		DeepLabv3 Plus	0.9253	0.8932	0.9244	0.9361
Datasets	Liver	ETU-Net(Ours)	**0.9533**	**0.9874**	**0.9934**	**0.9939**

The highest-performing results in the table are shown in bold.

### 4.6. Visualization of prediction results of ETU-Net model

**Figure 12:**
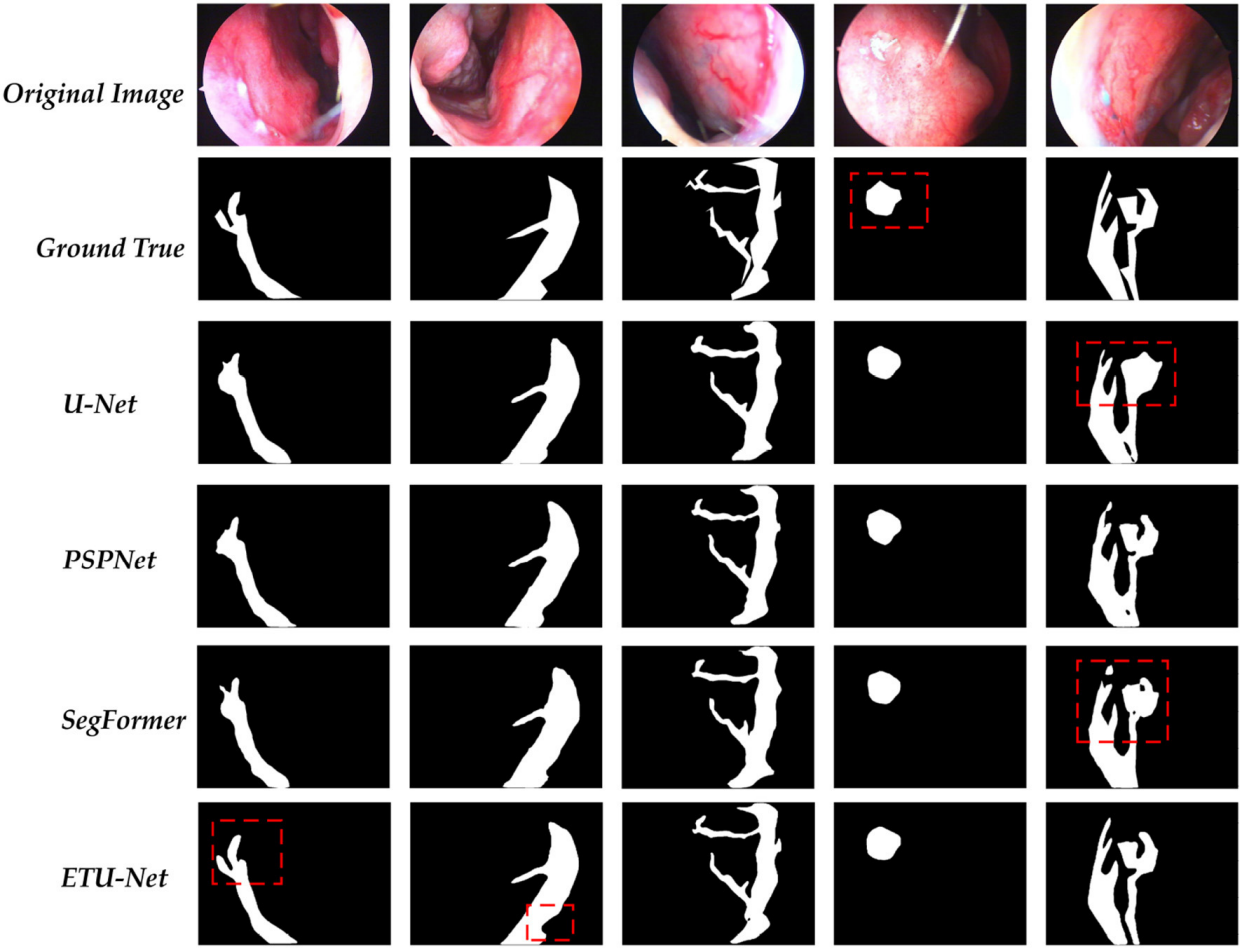
A visualization of the predictions for each model. Among them, the part marked by the dotted red box needs special attention for it can reflect the differences between each model in the prediction process.

In this section, we provide the visualization of the prediction results of the ETU-Net model, as shown in Figure 12. We compared the original image, the real label, and the prediction results of the four model models. These four images are all from the test set. For the parts that need special attention, we use red dotted boxes to highlight them. Through the comparison of the first column and the second column, it can be clearly seen that the ETU-Net model has a higher level of segmentation refinement than the U-Net and PSPNet models. Due to the lack of local scale awareness in the model structure and the insufficient receptive domain of the feature map, which causes the loss of local detail information, the prediction results of U-Net and PSPNet are typically circular. In the results of the fourth column, the ETU-Net models prediction is more in line with the real bleeding area, covering almost all the pixels in the area, and is even better than manual labeling. In the results of the fifth column, the role of context information in segmentation be clearly reflected. Taking U-Net as an example, The result predicted by the model in the upper branch on the right is approximately a circle. Both PSPNet and SegFormer think that there is only one main stem bleeding in this picture, while the ETU-Net model distinguishes that there are two branches here, and the direction of the tendril is well- judged. Thus it can be seen that the ETU-Net model proposed in this paper has good context awareness as well as local attention acquisition and has excellent performance for extremely fine medical segmentation tasks.

## 5. Conclusions

In this research, we present an ETU-Net model based on the Encoder-Decoder structure to solve the endoscopic epistaxis image segmentation challenge and assist physicians in distinguish bleeding area and abnormal vessels in practice. Because medical images are regular, AI algorithms may mine data attributes, learn, and eventually provide a model that can be deployed and applied.

We first proposed a Nasal Bleeding dataset as an evaluation basis. The dataset was presented for the first time in this field and was collected and labeled by doctors with years of clinical experience. Secondly, we use the Down Block module to strengthen the image feature extraction during the down-sampling process, use the group convolution to improve the extraction efficiency, and use the SE Block to introduce the attention mechanism; SC-Transformer reflects that our model uses CNN combined with Transformer architecture. Ordinary deep learning models have limitations in the receptive field in the image. It is challenging for them to capture adequate contextual feature information with high efficiency, so it will cause confusion between the bleeding area and the surrounding normal vessels and a lack of generalization at different scales, which makes it difficult to judge the direction of bleeding branches. As a result, we investigate the U-Net skip connection, which can deliver shallow feature map information to the decoder. However, the simple skip connection has limitations in that because it only evaluates correlation between the same levels, resulting in semantic gaps between various layers, hence we use SC-Transformer for multi-scale feature fusion. Transformer has an efficient multi-head self-attention mechanism and strong sequence modeling ability. It divides the two-dimensional information of the image into blocks and maps them into a linear embedding sequence, thereby converting it into one-dimensional sequence information to complete the process of feature fusion. Therefore, it is more appropriate to use it to design skip connections and achieve multi-scale fusion, which can make up for the lack of convolution. In the process of upsampling, we propose an attention Up Attention Block module based on channel and space, which converts the result of the Transformer into data that can be processed by convolution, gradually restores the image size and do the final for each pixel classification. In summary, the ETU-Net model is a novel U-type model combining CNN with Transformer. It has good context awareness and multi-scale feature fusion capabilities and can effectively identify global and local information.

Furthermore, we conducted a step-by-step analysis of the models superiority, conducted experiments on dataset semantic labeling, migration learning, and ablation of each module, and conducted domain migration experiments on DRIVE, Kvasir, and Liver datasets, including datasets of different scales and images collected by various devices, all of which demonstrate that our model can play a role in multiple fields. In particular, we delved into the role of the Transformer in ETU-Net. By comparing the performance of ETU-Net and traditional pure Transformer-based semantic segmentation networks such as SegFormer in Table 1, we found that several metrics of ETU-Net surpass those of SegFormer. Considering the substantial computational cost of pure Transformer architectures, we believe that adopting the ETU-Net convolutional fusion Transformer architecture offers more robust performance. Moreover, ablation experiments in Table 4 reveal that the Transformer, when employed as skip connections, significantly contributes to the success of ETU-Net. Due to the Transformer's capability for long-range sequence modeling, especially its multi-head attention mechanism, the model is guided to focus on contextual information. When replacing the SC-Transformer in ETU-Net, several metrics decline. Consequently, we argue that incorporating a Transformer at appropriate positions within the network is effective, and for small-scale medical imaging datasets, employing the Transformer to guide convolutional modules for segmentation between the encoder and decoder is a promising approach.

Our work shows that deep learning technology can learn and acquire knowledge in the data to obtain the same or higher level of accuracy of epistaxis image recognition as professional, help to reduce the complications caused by inexperienced operators and address the problems of lack of senior doctors. Next, we will explore the feasible deployment of ETU-Net and conduct clinical research to verify its effectiveness in assisting segmentation in clinical practice, so as to truly improve peoples health.

## Data availability statement

The datasets presented in this study can be found in online repositories. The names of the repository/repositories and accession number(s) can be found at: https://github.com/colorfulandcjy0806/ETU-Net.

## Ethics statement

Ethical review and approval was not required for the study on human participants in accordance with the local legislation and institutional requirements. The patients/participants provided their written informed consent to participate in this study. Written informed consent was obtained from the individual(s) for the publication of any potentially identifiable images or data included in this article.

## Author contributions

JC, QL, JLi, and YX: conceptualization and writing—review and editing. QL, XL, JLi, and YX: data curation. JC, QL, ZW, ML, and JLiu: formal analysis. JLi and YX: funding acquisition, project administration, resources, and supervision. JC, QL, ZW, XL, ML, and HC: investigation. JC, QL, ZW, XL, ML, HC, JLiu, and JLi: methodology. HC and JLiu: software. JC, QL, and JLi: validation. JC: visualization. JC, QL, ZW, and ML: writing—original draft. All authors contributed to the article and approved the submitted version.
